# A two-phase low-dose ACD-A strategy overcomes hypocalcemia during peripheral blood stem cell collection: a randomized controlled study

**DOI:** 10.3389/fonc.2026.1909033

**Published:** 2026-08-26

**Authors:** Zhangbiao Long, Yutong Jin, Dinghui Zhao, Yuxin Li, Zhengfeng Hou, Min Ruan, Jian Hong, Jifei Dai, Xinglin Liang, Qingsheng Li, Jian Ge

**Affiliations:** 1 Department of Hematology, The First Affiliated Hospital of Anhui Medical University, Hefei, China; 2 School of Pharmacy, Anhui University of Chinese Medicine, Hefei, China

**Keywords:** ACD-A, hypocalcemia, low dose, platelet aggregation, stem cell collection

## Abstract

**Background:**

Citrate anticoagulation during peripheral blood stem cell (PBSC) collection frequently causes hypocalcemia. Conventional fixed-ratio protocols frequently employ prophylactic calcium supplementation in many centers; however, they are associated with a high citrate burden and have been linked to platelet aggregation in some reports. This randomized controlled study evaluated a two-phase low-dose Acid Citrate Dextrose formula A (ACD-A) strategy designed to reduce hypocalcemia without compromising collection efficiency.

**Methods:**

Consecutive donors undergoing PBSC collection were randomly assigned to two groups. The Control group (n=22) received a blood-to-ACD-A ratio of 10-12:1 with prophylactic intravenous calcium. The Low ACD-A group (n=21) received initial loading phase at a ratio of 10-12:1 until 1 mL/kg of ACD-A was infused, followed by maintenance at 25:1 without prophylactic calcium. The primary outcome was the incidence of hypocalcemia-related symptoms. Secondary outcomes included collection time, ACD-A intake, CD34+ cell yield, and platelet aggregation.

**Results:**

The incidence of hypocalcemia-related symptoms was significantly lower in the Low ACD-A group than in the Control group (14% vs. 73%, *P* = 0.0002). Collection time was shorter (170.8 vs. 199.3 min, *P* = 0.0029), and ACD-A intake was substantially reduced (6.10 vs. 12.96 mL/kg, *P* < 0.0001). No significant differences were observed in CD34+ cell yield, enrichment ratio, yield per liter processed, or changes in ionized calcium levels between groups. Mild platelet aggregation occurred less frequently in the Low ACD-A group (9.5% vs. 41%, *P* = 0.0339).

**Conclusions:**

A two-phase low-dose ACD-A strategy significantly reduces hypocalcemia-related symptoms, shortens collection time, and decreases platelet aggregation without compromising stem cell yield or product quality. This simple modification represents a meaningful improvement over conventional citrate protocols for PBSC collection.

## 1 Introduction

Peripheral blood stem cell collection is the main method for harvesting hematopoietic stem cells. It is widely used in both autologous and allogeneic transplantation (1). The procedure involves stem cell mobilization, peripheral blood collection, mononuclear cell separation, and return of uncollected blood components (2). Compared with bone marrow harvest, this approach is simpler, more efficient, and avoids anesthesia and discomfort. It is now the standard method for stem cell collection.

The main adverse reaction during peripheral blood stem cell collection is hypocalcemia. It is caused by the widely used Acid Citrate Dextrose formula A (ACD-A), which chelates calcium in the blood (3). Conventional protocols recommend a blood-to-ACD-A ratio of 10-12:1. This results in a large amount of citrate infusion. Common symptoms include perioral numbness, tetany, nausea and vomiting. Some donors even develop arrhythmia, requiring interruption of collection or calcium supplementation (4–6). Recent large-scale registry data indicate that hypocalcemia remains one of the most frequently reported complications during apheresis procedures, underscoring the persistent clinical challenge (7). Hypocalcemia is common and remains a key complication to address during stem cell collection.

Some researchers have addressed this issue with prophylactic calcium supplementation, given either intravenously or orally. This approach reduces the incidence and severity of hypocalcemia-related symptoms and allows collection to proceed smoothly (8–10). However, prophylactic calcium supplementation has limitations. On one hand, high-dose citrate chelates calcium for anticoagulation; on the other hand, calcium is given prophylactically. This contradictory practice keeps donors in a state of high-level imbalance. Moreover, activated platelets have been anecdotally observed in the extracorporeal circuit and collection bag in some cases when prophylactic calcium is used, potentially related to the fluctuating calcium environment during citrate anticoagulation (6, 9).

Based on our clinical experience, we found that switching to low-dose citrate maintenance after adequate anticoagulation preserves anticoagulant efficacy while avoiding the impact of sustained high-dose citrate on blood calcium. Therefore, we designed a prospective, single-center, randomized controlled clinical study. In this study, we adopted this anticoagulation strategy for peripheral blood stem cell collection. We assessed the occurrence of hypocalcemia and other adverse reactions during collection and examined platelet aggregation in the collected product, aiming to identify an improved anticoagulation strategy for stem cell collection.

## 2 Methods

### 2.1 Donors and study design

**Figure 1:**
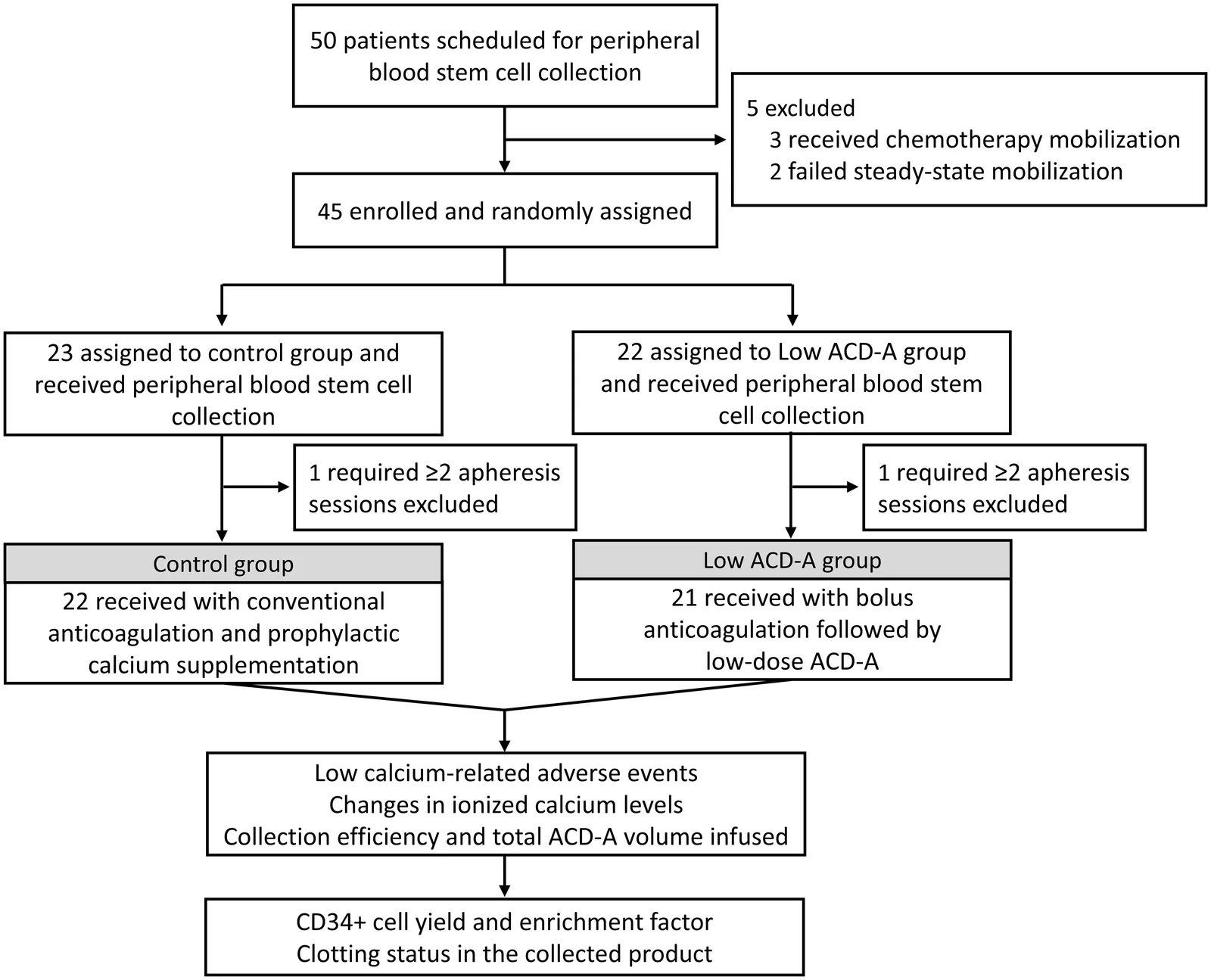
Flow diagram of donor enrolment and study groups. A total of 43 participants undergoing peripheral blood stem cell collection were enrolled and divided into the Control group (standard ACD-A with prophylactic intravenous calcium) and the Low ACD-A group (initial loading phase of ACD-A followed by maintenance at a blood to ACD-A ratio of 25:1 without prophylactic calcium). ACD-A, Acid Citrate Dextrose formula A.

This was a prospective, single-center, randomized controlled study. A total of 50 donors scheduled for peripheral blood stem cell collection at the First Affiliated Hospital of Anhui Medical University between July 2025 and June 2026 were initially screened, including patients with multiple myeloma or lymphoma scheduled for autologous transplantation and healthy donors for allogeneic transplantation. Inclusion criteria were: (1) age ≥ 15 years; (2) diagnosis of multiple myeloma or lymphoma scheduled for autologous PBSC collection, or healthy donor scheduled for allogeneic PBSC collection; (3) adequate organ function for apheresis. Exclusion criteria were: (1) receipt of chemotherapy for stem cell mobilization; (2) failure of steady-state mobilization; (3) requirement for more than two apheresis sessions; (4) active infection or unstable cardiovascular disease; (5) severe renal impairment (serum creatinine > 176.8 μmol/L or requiring dialysis) or severe hepatic impairment (ALT/AST > 3 times the upper limit of normal). Seven donors were excluded: three who received chemotherapy for mobilization, two who failed steady-state mobilization, and two who required more than two apheresis sessions. The remaining 43 donors were randomly assigned to two groups according to the anticoagulation strategy used during collection: the Control group (n = 22) received conventional ACD-A anticoagulation with prophylactic intravenous calcium, and the Low ACD-A group (n = 21) received a low-dose ACD-A protocol without prophylactic calcium (**Figure 1**). The study complied with the Declaration of Helsinki and was approved by the Ethics Committee of the First Affiliated Hospital of Anhui Medical University. All participants provided written informed consent.

### 2.2 Baseline characteristics

All donors underwent baseline assessment before stem cell collection, including age, sex, height, weight, BMI, and performance status assessed using the Eastern Cooperative Oncology Group (ECOG) performance status scale (all donors had ECOG 0 or 1). Laboratory tests included complete blood count, biochemistry (alanine aminotransferase [ALT], aspartate aminotransferase [AST], creatinine [Cr], blood urea), CD34+ cell count, white blood cell count, and ionized calcium level. These data were used to evaluate indications and contraindications for stem cell collection and to serve as baseline for comparison with post-collection data.

### 2.3 Peripheral blood stem cell collection procedure

All donors underwent steady-state mobilization with recombinant human granulocyte colony-stimulating factor at 10 μg/kg per day for 5 days. CD34+ cell count was measured one day before collection, and plerixafor was given if the count was below 20/μL. Collection was performed using the autoMNC mode on the COM.TEC device (Fresenius-Kabi, Germany) or the continuous mononuclear cell collection mode on the Spectra Optia device (Terumo BCT, USA) (11, 12). All procedures were performed via a double-lumen central venous catheter (CVC) in the internal jugular or subclavian vein.

ACD-A was used as the anticoagulant (7). In the Control group, the blood-to-ACD-A ratio was 10-12:1. In the Low ACD-A group, the same ratio (10-12:1) was used as an initial loading phase until a total ACD-A volume of 1 mL/kg was infused (this refers to the volume of ACD-A solution administered by the machine into the extracorporeal circuit, corresponding to the citrate load returned to the donor), after which the ratio was adjusted to 25:1 for maintenance. On the Fresenius COM.TEC device, the maximum programmable blood-to-ACD-A ratio is 25:1, and values above this cannot be set; on the Terumo Spectra Optia device, the upper limit can be manually adjusted up to 50:1. In our Low ACD-A group, we uniformly adopted a maintenance ratio of 25:1 to standardize the protocol across both devices and enhance clinical applicability. The Control group received intravenous calcium supplementation to prevent hypocalcemia: calcium gluconate 10% solution (100 mg/mL), given as an initial slow intravenous bolus of 1 g (10 mL) followed by a continuous maintenance infusion of 1 g/hour via an infusion pump, adapted from the protocol described by Hegde et al. (9). The Low ACD-A group did not receive prophylactic calcium. When grade 1 hypocalcemia symptoms occurred, oral calcium gluconate was given. For higher-grade symptoms, intravenous calcium was administered, the collection speed was reduced, or the procedure was temporarily suspended if severe. All donors completed stem cell collection within one day. The collection targets were: ≥ 2 × 10^6^ CD34+ cells/kg for autologous donors and ≥ 4 × 10^6^ CD34+ cells/kg (recipient body weight) for allogeneic donors (13).

### 2.4 Assessment of hypocalcemia

Hypocalcemia symptoms were graded according to Bolan et al. (14): grade 0, asymptomatic; grade 1, mild numbness; grade 2, moderate numbness; grade 3, moderate numbness with nausea, nervousness, or anxiety; grade 4, arrhythmia, tetany, or seizures. Symptoms were assessed by experienced attending physicians, who provided appropriate management. Ionized calcium levels were measured before and after stem cell collection using a direct ion-selective electrode (ISE) method on a GEM Premier 3500 blood gas analyzer (Instrumentation Laboratory, Bedford, MA, USA) to evaluate changes.

### 2.5 Procedure parameters

During stem cell collection, the following parameters were recorded: total blood volume processed, number of cycles, final product volume, ACD-A intake, collection speed, and processed plasma volume. Adverse reactions other than hypocalcemia were also documented. The extracorporeal circuit (including tubing, centrifuge chamber via observation window, and collection bag) was inspected for the presence of platelet aggregation at three standardized time points: (1) after the initial 10 minutes of circulation, (2) at the midpoint of the procedure, and (3) immediately after completion upon disconnection of the disposable set. Aggregation was recorded as “present” when visible white/off-white clumps or fibrin strands were identified.

### 2.6 Assessment of collected product

After collection, CD34+ cell count and white blood cell count were measured. We assessed whether the CD34+ cell yield met the target (≥ 2 × 10^6^ CD34+ cells/kg for autologous donors; ≥ 4 × 10^6^ CD34+ cells/kg for allogeneic donors). The enrichment ratio of CD34+ cells (CD34+ in final product divided by CD34+ in peripheral blood) was calculated. The CD34+ yield per liter of blood processed was calculated as: (CD34+ concentration in final product × final product volume) ÷ (total blood volume processed in liters), where total blood volume processed refers to the whole donor blood volume (excluding ACD-A) as recorded by the apheresis device. The collection bag was also inspected for platelet aggregation as described in Section 2.5.

### 2.7 Statistical analysis

Continuous data following a normal distribution were expressed as mean ± standard deviation; those not following a normal distribution were expressed as median and interquartile range. Some continuous variables were converted into categorical variables based on clinical relevance. Categorical data were expressed as frequencies and percentages n (%). For comparisons between two groups, continuous variables were analyzed using the independent samples *t*-test or Mann-Whitney *U* test, and categorical variables were analyzed using the Pearson chi-square test or Fisher’s exact test. A two-sided *P* < 0.05 was considered statistically significant.

Sample size calculation: Based on our pilot data, we expected a reduction in the incidence of hypocalcemia-related symptoms from 60% in the Control group to 20% in the Low ACD-A group. Using a two-sided chi-square test with a significance level (α) of 0.05 and a statistical power (1-β) of 0.80, we calculated that approximately 19 donors per group were needed. Considering a potential 10% dropout rate, we planned to enroll 21–22 donors per group. Over the study period, 43 donors were ultimately randomized, which exceeded the required sample size for the primary endpoint. This calculation was performed using PASS software (version 15.0, NCSS, LLC, Kaysville, UT, USA). All statistical analyses were performed using GraphPad Prism version 9.5.0 (GraphPad Software, San Diego, CA, USA).

## 3 Results

### 3.1 Baseline characteristics

A total of 43 donors were enrolled, including 33 patients for autologous transplantation and 10 healthy donors for allogeneic transplantation. Among the autologous patients, 26 had multiple myeloma and 7 had lymphoma (1 DLBCL, 2 MCL, 1 NK/T-cell lymphoma, 1 ALAL, 1 HL, and 1 unspecified NHL). All autologous donors had achieved complete remission or partial remission. None of the MM donors had received Daratumumab prior to collection. According to the anticoagulation strategy, 22 donors were assigned to the Control group and 21 to the Low ACD-A group. The mean age was 48.33 ± 15.47 years, with 30 males and 13 females. No significant differences were observed between the two groups in age, sex, height, weight, BMI, or performance status (*P* > 0.05).

In the Control group, there were 17 patients with multiple myeloma, 1 with lymphoma, and 4 healthy donors. In the Low ACD-A group, there were 9 with multiple myeloma, 6 with lymphoma, and 6 healthy donors (*P* = 0.0188 for disease type distribution). Regarding the apheresis device, 13 donors in the Control group and 13 in the Low ACD-A group used COM.TEC; 9 in the Control group and 8 in the Low ACD-A group used Spectra Optia (*P* > 0.05). No significant differences were observed in pre-collection CD34+ cell count, ionized calcium level, or liver/renal function parameters (P > 0.05) (**Table 1**).

**Table 1:** Baseline characteristics of donors undergoing peripheral blood stem cell collection.

Characteristics	Whole cohort	Control	Low-ACD	*P* value
Age (year)	48.33 ± 15.47	51.64 ± 14.37	44.86 ± 16.16	0.1533
Gender				
Male	30	16	14	0.7470
Female	13	6	7	
Height (cm)	168.30 ± 7.49	168.20 ± 7.02	168.40 ± 8.12	0.9473
Weight (kg)	69.64 ± 12.61	65.59 ± 12.67	69.69 ± 12.85	0.9797
BMI (kg/m^2^)	24.46 ± 3.36	24.44 ± 3.23	24.47 ± 3.57	0.9784
ECOG 0-1	43	22	21	>0.9999
SCT type				
autologous SCT	33	18	17	0.7222
allogeneic SCT	10	4	6	
Disorder type				
MM	26	17	9	0.0405
Lymphoma	7	1	6	
Healthy donor	10	4	6	
Apheresis device				
COM.TEC	26	13	13	>0.9999
Spectra Optia	17	9	8	
CD34+cells (/μL)	66.40 ± 36.90	67.95 ± 31.60	64.76 ± 42.48	0.7805
WBC (×10^9^/L)	43.39 ± 14.89	46.94 ± 15.97	39.68 ± 13.01	0.1112
Ionized calcium (mmol/L)	1.19 ± 0.09	1.19 ± 0.10	1.19 ± 0.08	0.9899
ALT (U/L)	20.77 ± 11.72	18.68 ± 9.38	22.95 ± 13.65	0.2369
AST (U/L)	21.77 ± 9.64	20.55 ± 5.43	23.05 ± 12.67	0.4012
Creatinine (μmol/L)	81.43 ± 40.09	83.36 ± 41.94	79.40 ± 38.99	0.7499
Urea (mmol/L)	6.31 ± 2.88	5.91 ± 2.57	6.69 ± 3.16	0.3835
PT (s)	12.58 ± 0.81	12.80 ± 0.63	12.36 ± 0.92	0.0729
APTT (s)	33.51 ± 4.26	34.00 ± 3.98	33.00 ± 4.57	0.4476

Data are presented as mean ± SD or *n* (%). ACD-A, acid citrate dextrose solution A; BMI, body mass index; ECOG, Eastern Cooperative Oncology Group; MM, multiple myeloma; SCT, stem cell transplantation; WBC, white blood cell count; ALT, alanine aminotransferase; AST, aspartate aminotransferase; Cr, creatinine; PT, prothrombin time; APTT, activated partial thromboplastin time. *P* values indicate statistical differences between the Control group and the Low ACD-A group.

### 3.2 Hypocalcemia-related symptoms and changes in ionized calcium levels

**Figure 2:**
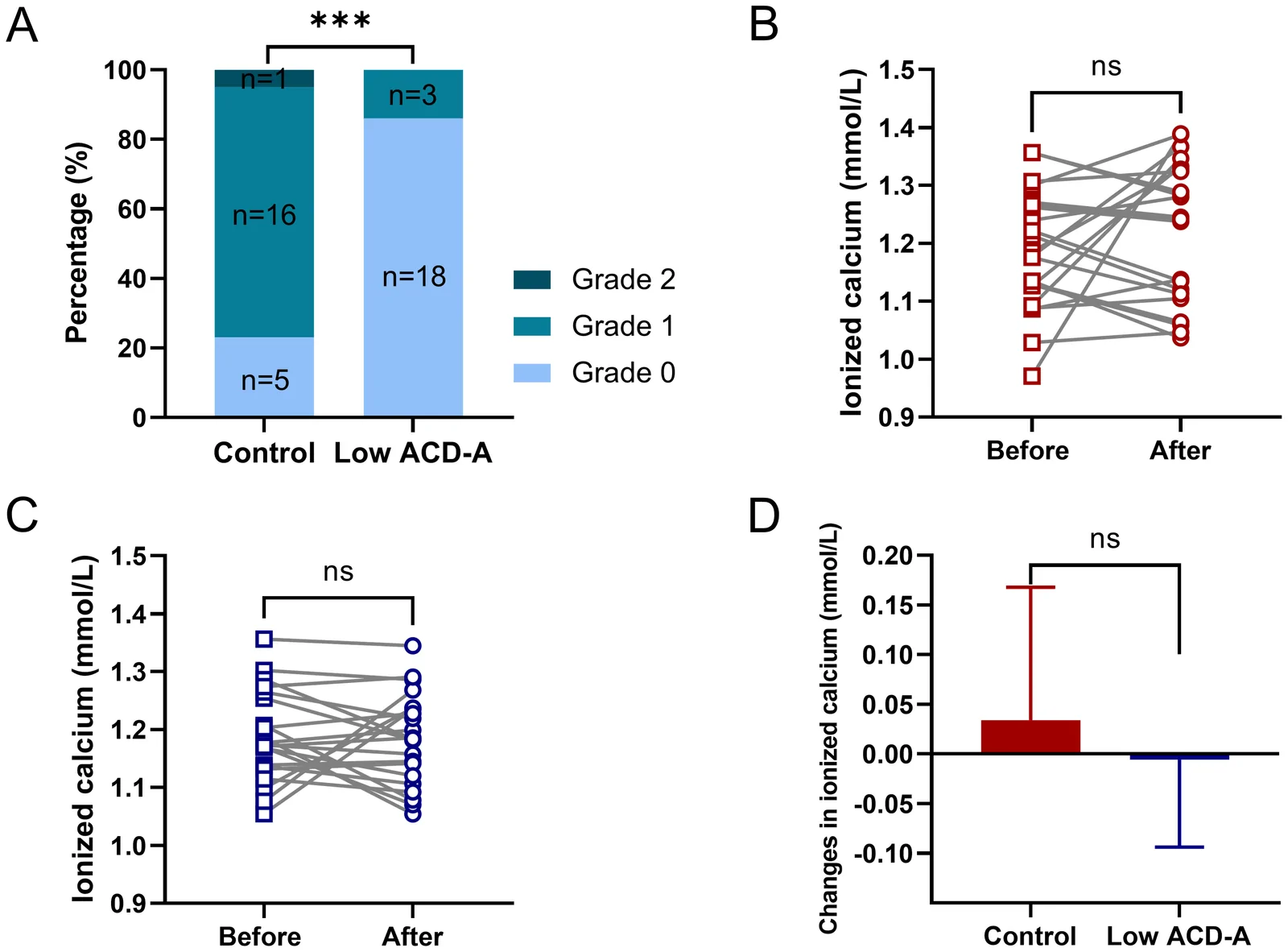
Hypocalcemia-related symptoms and changes in ionized calcium levels. **(A)** Incidence and severity of hypocalcemia-related symptoms in the Control group and Low ACD-A group. **(B)** Ionized calcium levels before and after stem cell collection in the Control group. **(C)** Ionized calcium levels before and after stem cell collection in the Low ACD-A group. **(D)** Comparison of the change in ionized calcium levels (post-collection minus pre-collection) between the two groups. Data are presented as mean ± SD. ***P < 0.001; NS, not significant.

A total of 20 donors experienced hypocalcemia-related symptoms during collection. In the Control group, 16 donors had grade 1 symptoms and 1 had grade 2 symptoms, with no grade 3 or higher symptoms. In the Low ACD-A group, 3 donors had grade 1 symptoms. The incidence was significantly lower in the Low ACD-A group than in the Control group (*P* = 0.0002) (**Figure 2A**). No unexpected adverse events occurred during the procedure.

Ionized calcium levels were measured before and after collection. In the Control group, there was no significant change in ionized calcium levels (**Figure 2B**). Similarly, in the Low ACD-A group, no significant change was observed (*P* > 0.05) (**Figure 2C**). The change in ionized calcium (post-collection minus pre-collection) was 0.034 ± 0.134mmol/L in the Control group and -0.006 ± 0.09 mmol/L in the Low ACD-A group, with no significant difference between the two groups (*P* > 0.05) (**Figure 2D**).

### 3.3 Procedure parameters and ACD-A intake

**Figure 3:**
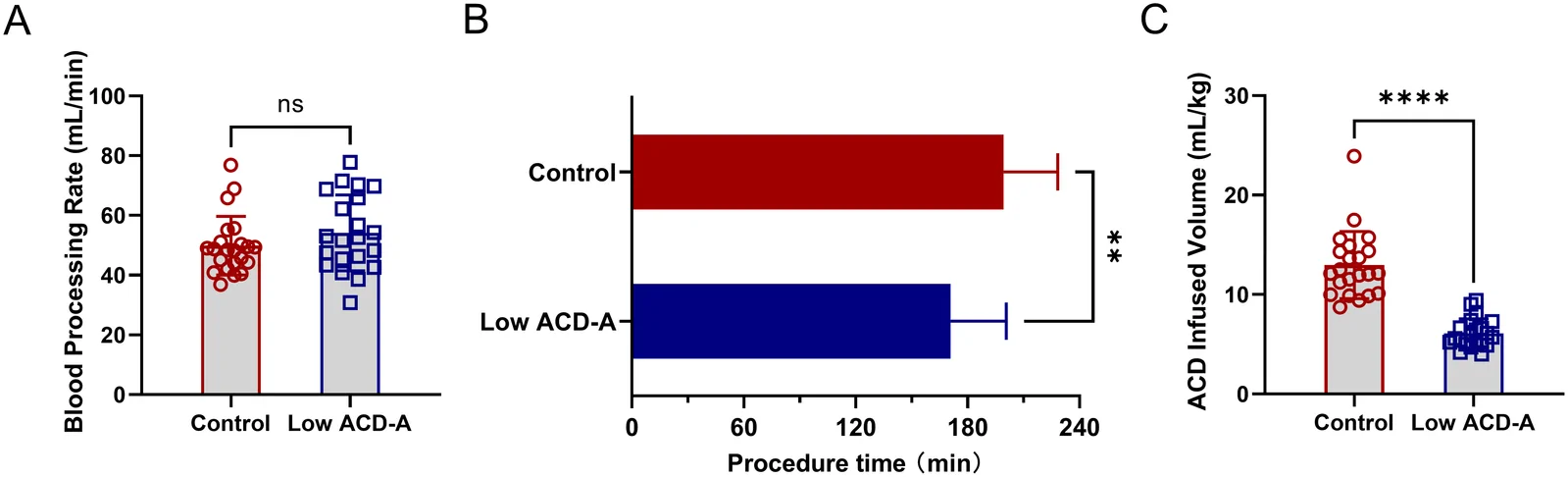
Procedure parameters during stem cell collection. **(A)** Whole blood processing flow rate was comparable between the two groups (NS). **(B)** The Low ACD-A group had a significantly shorter collection time than the Control group (*P* = 0.0029). **(C)** ACD-A intake per kilogram body weight was significantly lower in the Low ACD-A group (*P* < 0.0001). Data are presented as mean ± SD. **P < 0.01; ****P < 0.0001; NS, not significant.

During collection, the whole blood processing flow rate was 49.88 ± 9.81mL/min in the Control group and 54.20 ± 12.71 mL/min in the Low ACD-A group. The Low ACD-A group had a faster flow rate, but the difference was not statistically significant (*P* > 0.05) (**Figure 3A**). Collection time was significantly shorter in the Low ACD-A group (170.8 ± 29.93 min *vs.* 199.3 ± 29.00 min, *P* = 0.0029) (**Figure 3B**). ACD-A intake was also significantly lower in the Low ACD-A group (6.10 ± 1.47 mL/kg *vs.* 12.96 ± 3.37 mL/kg, *P* < 0.0001) (**Figure 3C**). Detailed procedural parameters, including processed volume, product volume, and device-specific data, are presented in **Table 2**.

**Table 2:** Procedural parameters during stem cell collection.

Parameter	Control	Low-ACD	*P* value
Total whole blood processed (mL)	9792 ± 1561	9167 ± 2269	0.2973
Procedure time (min)	199.3 ± 29.00	170.8 ± 29.93	0.0029
ACD-A intake (mL/kg)	12.96 ± 3.37	6.10 ± 1.47	<0.0001
Final product volume (mL)	253.7 ± 48.92	217.2 ± 50.95	0.0212
Average inlet flow rate (mL/min)	49.88 ± 9.81	54.20 ± 12.71	0.2179
COM.TEC	n=13	n=13	
Total whole blood processed (mL)	9126 ± 1997	9871 ± 1179	0.2580
Procedure time (min)	202.5 ± 35.57	170.8 ± 25.59	0.0154
Spectra Optia	n=9	n=8	
Total whole blood processed (mL)	9677 ± 2071	9235 ± 2803	0.7142
Procedure time (min)	194.6 ± 16.35	170.8 ± 37.91	0.1064
CD34+ yield per liter processed (×10^6^/L)	39.19 ± 19.45	42.68 ± 32.85	0.6722

Data are presented as mean ± SD. ACD-A, Acid Citrate Dextrose formula A. Total whole blood processed refers to the volume of donor blood recorded by the apheresis device, excluding the ACD-A volume.

### 3.4 Stem cell yield, enrichment ratio, and platelet aggregation

**Figure 4:**
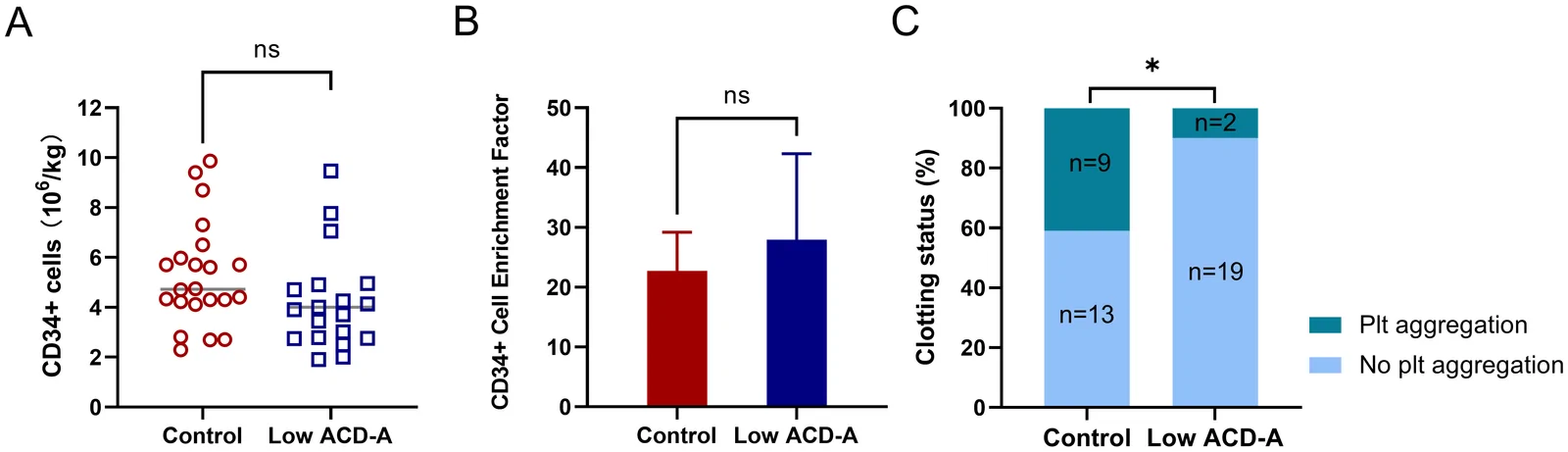
Stem cell yield, enrichment ratio, and platelet aggregation. **(A)** CD34+ cell yield was comparable between the two groups (NS). **(B)** Enrichment ratio of CD34+ cells was higher in the Low ACD-A group, but the difference was not statistically significant (NS). **(C)** Mild platelet aggregation occurred less frequently in the Low ACD-A group than in the Control group (*P* = 0.0339). Data are presented as mean ± SD. *P < 0.05; NS, not significant.

All donors achieved the collection target. CD34+ cell yield was 5.27 ± 2.09× 10^6^/kg in the Control group and 5.10 ± 3.39 × 10^6^/kg in the Low ACD-A group, with no significant difference (*P* > 0.05) (**Figure 4A**). The enrichment ratio (CD34+ in final product divided by CD34+ in peripheral blood) was 22.73 ± 6.45 in the Control group and 27.95 ± 14.34 in the Low ACD-A group. The Low ACD-A group had a higher enrichment ratio, but the difference was not statistically significant (*P* > 0.05) (**Figure 4B**). Mild platelet aggregation was observed in 9/22 (41%) of the Control group and 2/21 (9.5%) of the Low ACD-A group, representing a significantly lower incidence in the Low ACD-A group (*P* = 0.0339) (**Figure 4C**).

## 4 Discussion

The aim of this study was to identify an optimal anticoagulation strategy that reduces adverse reactions during stem cell collection without compromising CD34+ yield per liter of blood processed or overall product quality. To minimize bias, we consecutively enrolled all donors who underwent stem cell collection at our center over one year. Notably, among the 50 initially screened donors, 47 received steady-state mobilization to reduce mobilization-related adverse reactions. The success rate of steady-state mobilization at our center was high, with 91% (43/47) of donors achieving adequate stem cell yield in a single collection. This rate is higher than those reported in previous studies (15–17). The timing of stem cell collection was carefully selected: patients with multiple myeloma or lymphoma undergoing autologous transplantation were scheduled for collection after 2–4 cycles of chemotherapy when they had achieved complete or partial remission. Mobilization was also optimized by measuring CD34+ cell count on day 4 of steady-state mobilization and administering plerixafor if the count was below 20/mL (for autologous donors only) (18, 19), an approach that resulted in high-quality mobilization in most donors, with only two donors experiencing mobilization failure and two requiring more than two apheresis sessions. Furthermore, stem cell collection at our center is an experience-based procedure; throughout the process, we adjusted parameters manually based on the collected product to obtain a high-quality final product. To ensure homogeneity between study groups, donors who received chemotherapy for mobilization, those who failed mobilization, and those who required more than two apheresis sessions were excluded before randomization.

In clinical practice, we found that after adequate anticoagulation with a bolus of ACD-A, switching to low-dose ACD-A maintenance did not result in significant platelet aggregation in the apheresis line or collection bag. Compared with the blood-to-ACD-A ratio of 10-15:1 recommended by the Fresenius COM.TEC and Terumo Spectra Optia devices, our strategy used less anticoagulant, reduced the impact on the donor’s internal environment, and better followed the “less is more” principle.

We carefully considered how to implement this two-phase, low-dose anticoagulation strategy. The initial phase used the same anticoagulant concentration as the Control group to ensure adequate anticoagulation and prevent platelet aggregation. After infusing 1 mL/kg of ACD-A, we switched to a maintenance phase with a blood-to-ACD-A ratio of 25:1, which is substantially lower than the conventional protocol. Further reduction of the initial bolus might increase platelet aggregation risk, while increasing the maintenance ratio above 25:1 warrants further investigation. The 25:1 ratio was selected as it is the upper limit allowed by the Fresenius COM.TEC device, and we uniformly adopted this concentration to enhance the clinical applicability of our study.

The prophylactic intravenous calcium regimen used in the Control group (calcium gluconate 10%, 1 g bolus followed by 1 g/hour continuous infusion) was adapted from the protocol published by Hegde et al. (9). This regimen was chosen to achieve rapid stabilization of ionized calcium at the onset of the procedure (when citrate load is highest) and maintain this stability throughout the collection. However, the 72.7% incidence of hypocalcemia symptoms in the Control group, despite prophylaxis, warrants discussion. As described in the Methods section, symptoms were assessed through active, structured questioning of donors at regular intervals, rather than passive reporting. The classic study by Bolan et al. (14) demonstrated that ionized calcium levels decreased by up to 33% during conventional ACD-A anticoagulation in a dose-dependent manner, and that donor symptoms became more severe as citrate infusion rates increased (14). In a recent randomized controlled trial by Abe et al. (2024) using active monitoring, the incidence of Grade ≥1 citrate-related symptoms was 42.9% (9/21) in the placebo group (9), confirming that active questioning yields higher detection rates than passive reporting. This explains why our observed incidence is higher than that reported in some registry-based studies (7) that rely on spontaneous reporting. Furthermore, the prophylactic calcium dose may not have been optimal for all individuals, given inter-individual variability in citrate metabolism and calcium kinetics. Importantly, this does not diminish the clinical value of our findings; rather, it emphasizes that even with prophylaxis, conventional high-citrate protocols frequently cause symptoms, and reducing the citrate load provides a superior solution.

Regarding baseline characteristics, the Control group and the Low ACD-A group were generally balanced, with the only statistically significant difference being disease type distribution. The Control group consisted mainly of patients with multiple myeloma and healthy donors, with only one lymphoma patient. Although the study was randomized, incomplete balance in baseline characteristics was observed, which may be attributed to the relatively small sample size; however, this did not affect the apheresis process or study outcomes. A highly significant difference was observed in hypocalcemia symptoms between the two groups. In the Control group, 73% (16/22) of donors developed hypocalcemia-related symptoms, compared with only 14% (3/21) in the Low ACD-A group. Symptoms were mild in both groups. Symptomatic donors in the Control group required speed reduction and accelerated calcium infusion, while the three symptomatic donors in the Low ACD-A group responded to oral calcium gluconate. Consequently, the Low ACD-A group had significantly fewer interruptions or slowdowns due to hypocalcemia, resulting in a smoother collection process and a shorter overall procedure time. We also measured ionized calcium levels before and after collection. The change was minimal in both groups, reflecting that both strategies maintained calcium stability: the Control group achieved a high-level balance between high-dose citrate chelation and prophylactic calcium supplementation, whereas the Low ACD-A group achieved a low-level balance by substantially reducing the amount of citrate used for chelation, thereby minimizing the impact on donors and avoiding the rapid fluctuations in ionized calcium that are more likely to trigger symptoms during the procedure.

This strategy has good applicability. It requires no additional infusion or manipulation and thus does not increase procedural difficulty. Moreover, unlike some studies that used other anticoagulants (20–22), we continued to use ACD-A, the most widely used anticoagulant in clinical practice, thereby reducing the risk of unexpected adverse reactions (7). Importantly, this study had a clear objective: to reduce hypocalcemia and other adverse reactions by modifying anticoagulation while minimizing the impact on collection efficiency and product quality. Our results show that the Low ACD-A strategy is simple and effective, reducing hypocalcemia without compromising collection efficiency.

This study has several limitations. First, although this was a randomized controlled study, it was single-center with a relatively small sample size, and baseline characteristics (particularly disease type distribution) were not fully balanced between groups due to the small sample size. Second, some enrolled donors had mild-to-moderate renal impairment (4 in the Control group and 3 in the Low ACD-A group); while G-CSF and plerixafor doses were adjusted accordingly per our institutional protocol, the small sample size precluded a formal subgroup analysis to assess whether renal function influenced citrate metabolism or the incidence of hypocalcemia. Third, this study included only two groups. The Control group received prophylactic calcium, which is consistent with most clinical practice; however, a three-arm design including a third group without prophylactic calcium might have allowed a clearer comparison of adverse reactions across different strategies. Fourth, we measured ionized calcium only before and after the procedure, not serially during the collection. Intra-procedural monitoring could have provided a more dynamic picture of calcium changes and better correlation with symptom onset. Fifth, platelet aggregation was assessed solely by visual inspection rather than objective quantitative platelet function assays (e.g., impedance aggregometry or flow cytometry). While visual inspection is the clinically actionable method used in routine practice, quantitative assays would provide more objective data. Sixth, our study excluded donors with severe renal or hepatic impairment, so our findings may not be generalizable to such populations where citrate metabolism could be substantially altered. Future multicenter, large-scale randomized controlled trials are needed to validate our findings and address these limitations.

In conclusion, in peripheral blood stem cell collection, a strategy of low-dose anticoagulation after adequate initial anticoagulation significantly reduces the incidence and severity of hypocalcemia. It shortens collection time by reducing interruptions due to adverse reactions. It does not compromise product quality, with minimal platelet aggregation in the collected product. This strategy is simple, easy to implement, and represents a practical innovation worthy of clinical application.

## Data Availability

The raw data supporting the conclusions of this article will be made available by the authors, without undue reservation.
